# Integrating DNA Barcoding Within an Orthogonal Approach for Herbal Product Authentication: A Narrative Review

**DOI:** 10.1002/pca.3466

**Published:** 2024-11-12

**Authors:** Nazia Nazar, Akanksha Saxena, Anu Sebastian, Adrian Slater, Velusamy Sundaresan, Tiziana Sgamma

**Affiliations:** ^1^ Biomolecular Technology Group, Leicester School of Allied Health Science, Faculty of Health and Life Sciences De Montfort University Leicester UK; ^2^ Plant Biology and Systematics CSIR—Central Institute of Medicinal and Aromatic Plants, Research Centre Bengaluru India; ^3^ Academy of Scientific and Innovative Research (AcSIR) Ghaziabad India

**Keywords:** adulteration, authentication, chemical analysis, DNA barcode, herbal products, metabarcoding, mini barcoding, orthogonal approach, quality assurance

## Abstract

**Introduction:**

Existing methods for morphological, organoleptic, and chemical authentication may not adequately ensure the accurate identification of plant species or guarantee safety. Herbal raw material authentication remains a major challenge in herbal medicine. Over the past decade, DNA barcoding, combined with an orthogonal approach integrating various testing methods for quality assurance, has emerged as a new trend in plant authentication.

**Objective:**

The review evaluates DNA barcoding and common alternative testing in plant‐related sectors to enhance quality assurance and accurate authentication.

**Method:**

Studies were selected based on their relevance to the identification, quality assurance, and safety of herbal products. Inclusion criteria were peer‐reviewed articles, systematic reviews, and relevant case studies from the last two decades focused on DNA barcoding, identification methods, and their applications. Exclusion criteria involved studies lacking empirical data, those not peer‐reviewed, or those unrelated to the main focus. This ensured the inclusion of high‐quality, pertinent sources while excluding less relevant studies.

**Results:**

An orthogonal approach refers to the use of multiple, independent methods that provide complementary information for more accurate plant identification and quality assurance. This reduces false positives or negatives by confirming results through different techniques, combining DNA barcoding with morphological analysis or chemical profiling. It enhances confidence in results, particularly in cases of potential adulteration or misidentification of plant materials.

**Conclusion:**

This study highlights the persistent challenges in assuring the quality, purity, and safety of plant materials. Additionally, it stresses the importance of incorporating DNA‐based authentication alongside traditional methods, to enhance plant material identification.

## 1 Introduction

Herbs and spices have long played a key role in both culinary and medicinal practices. With modern technology and scientific advances, secondary compounds plants produce have gained attention for their unique chemical properties, leading to their use in food, medicine, and cosmetics. The herbal and spice market has grown significantly in the past decade, driven by their functional properties and increased awareness of health benefits associated with herbal products [1, 2]. Additionally, the COVID‐19 pandemic has further accelerated the demand for herbal products, particularly those having immune‐boosting properties [3, 4]. Herbal plant species known for their antiviral properties from various genera such as *Citrus*, *Allium*, *Mentha*, *Ocimum*, and *Nigella* have become highly sought‐after for their anticipated potential health benefits [5, 6, 7, 8, 9, 10]. This trend is expected to continue, indicating a sustained and growing demand for herbal products in various industries [1, 11]. However, concerns regarding sustainability and quality control of herbal products need to be addressed to ensure ethical sourcing and safe usage.

**FIGURE 1:**
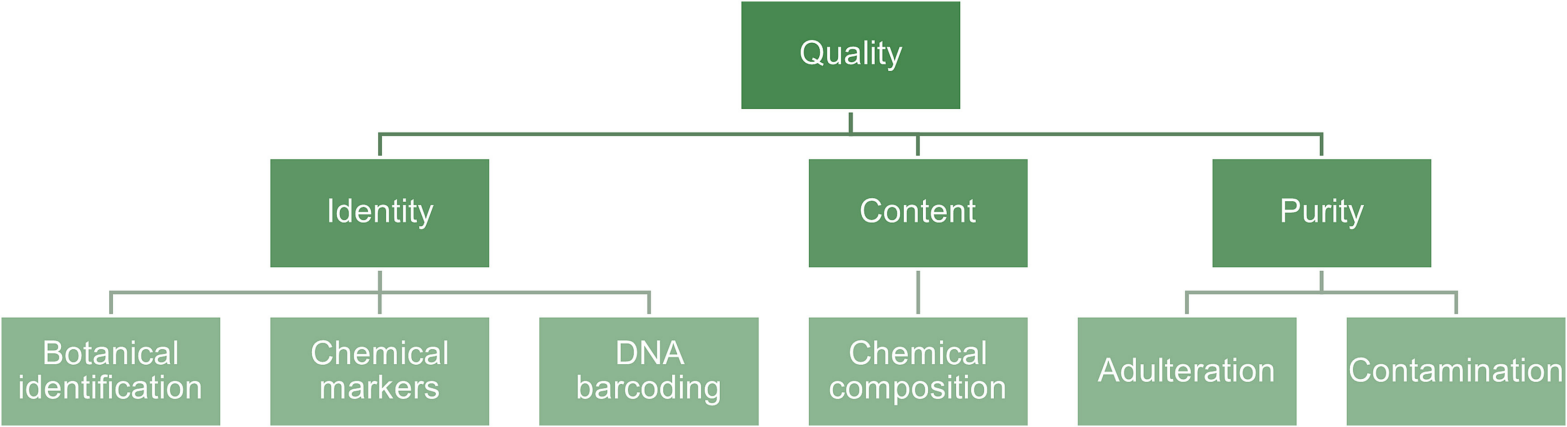
Overview of the authentication process in quality assurance.

With the globalization of the herbal market, the risk of raw materials being contaminated with adulterants or substitutes has increased, threatening patient safety and herbal efficacy [12, 13, 14, 15, 16]. Globally, several safety issues have arisen from incorrect or misleading authentication of medicinal herbs and their source plants. Authentication, a quality assurance process, verifies plant species, using methods like anatomical and morphological features, chemical analysis, DNA testing, or a combination of these methods [17, 18, 19, 20], as shown in Figure 1. DNA barcoding and chemical testing can also detect adulteration. In addition to species identification, chemical tests are also used to measure the concentration of specific compounds when chemical markers are of interest. The term authentication is commonly used in the context of ensuring the correct identity of plant‐based products. In contrast, identification specifically refers to determining the plant species, particularly before processing them for product development or research [21, 22, 23]. Therefore, proper identification and authentication are essential to ensure the safe and effective use of herbal products in modern medicine and health care [24, 25].

Despite the long history of herbal medicine usage, only a limited number of plant species have been extensively studied for their potential medical applications [26, 27]. Furthermore, herbal medicines are often complex mixtures, and their safety and efficacy often depend on their metabolite content. This represents a challenge not only when researchers want to evaluate the efficacy of bioactive compounds but also when they want to evaluate the authenticity of the botanicals [28, 29]. However, with the increasing understanding of both the traditional knowledge and scientifically documented benefits of herbs, consumers are now more inclined to explore herbal remedies as viable alternatives to conventional, mono‐substance‐based supplements and medicines. Recognizing this shift, the World Health Organization (WHO) has highlighted the growing acceptance of traditional medicine (TM) in national health systems [30]. Many countries are actively developing policies, enacting laws and regulations, and implementing frameworks to govern herbal medicines [31]. However, the rise of adulteration poses a significant concern to the herbal product industry. Although intentional adulteration is considered a deliberate malpractice, unintentional or accidental adulteration can also occur during the trade of raw materials. Some historical examples are given in Table 1. This review article will evaluate the state of the use of DNA barcoding and alternative/complementary testing in plant‐related sectors to enhance quality assurance and accurate identification.

**TABLE 1:** Examples of plant species, their adulterants, and associated effects.

Species name	Common name	Adulterants	Adulteration	Effects	References
*Stephania tetrandra*	Fang ji, Han Fang Ji	*Aristolochia fangchi*	Accidental	*Aristolochia* contains concentrations of aristolochic acid that can result in nephrotoxicity.	Tankeu *et al* [32]
*Hypericum perforatum*	St. John's Wort	*H*. *barbatum*, *H. hirsutum* , *H. undulatum* , and other species from genus *Hypericum*	Accidental and intentional	*H*. *perfotatum* is the most used herb for treating depression. Adulteration from the same genera may significantly reduce efficacy.	Booker *et al*. [33]; Sgamma *et al*. [34]
*Echinacea* spp.	Purple coneflower	*Parthenium integrifolium* , Helianthus spp., *Lespedeza capita*, *Eryngium aquaticum* , and *Rudbeckia nitida*	Accidental and intentional	Adulteration could decrease the safety, efficacy, and reliability of commercial *Echinacea* products.	Zhang *et al*. [35] Gafner *et al*. [35]
*Lavender angustifolia*	Lavender	*Lavandula* × *intermedia*	Intentional	Reduce the quality of Lavender oil.	Bejar [36]
*Sambucus* spp.	Elderberry	*Oryza sativa*	Intentional	Reduce health benefits to consumers.	ABC‐AHP‐NCNPR [37]
*Scrutellaria* spp.		*Teucrium* spp.	Accidental and intentional	Adulteration causes liver toxicity.	Foster [38]
*Nigella sativa*	Black seeds	With seeds of the same size and color and oils on sunflower and soybean.	Intentional	Reduce benefits to consumers.	Orhan [39]
*Origanum vulgare*	Oregano	*Cistus* spp., *Corylus avellana* , *Fragaria* spp., *Myrtus communis* , *Origanum majorana* , and *Thymus* spp.	Accidental and intentional	Effects on the quality of herbs and oil.	Black *et al*. [40]
*Withania somnifera*	Ashwagandha	Roots substituted with aerial parts	Intentional	Reduce the potency and therapeutic effectiveness of the herbal product, potentially diminishing its intended benefits.	Kumarsingh *et al*. [41]
*Curcuma longa*	Turmeric	*Curcuma* spp. and synthetic curcuminoids and color	Intentional	Published reports indicate a reduction in the efficacy of the herb as well as instances of lead toxicity.	Bejar [42]

## 2 Morphological‐Based Plant Species Identification and the Need for Complementary Methods

Morphological traits—both macroscopic and microscopic—have been extensively used in scientific investigation and botanical quality control [43, 44, 45, 46]. Macroscopic examination is based on shape, size, color, surface characteristics, and organoleptic elements, however, these characteristics can vary depending on environmental conditions and time of harvest [47, 48]. Microscopic examination analyzes structural, cellular, and molecular features of herbal products using various microscopy techniques [49, 50], as detailed in several pharmacopeias, notably the European, British, United States, and Chinese Pharmacopoeias. However, in the herbal plant sector, relying solely on morphological features can lead to incorrect identification of plant material, posing risks to consumer health [51]. Below are a few reported examples:

### 2.1 Case Studies

#### 2.1.1 Echinacea

**FIGURE 2:**
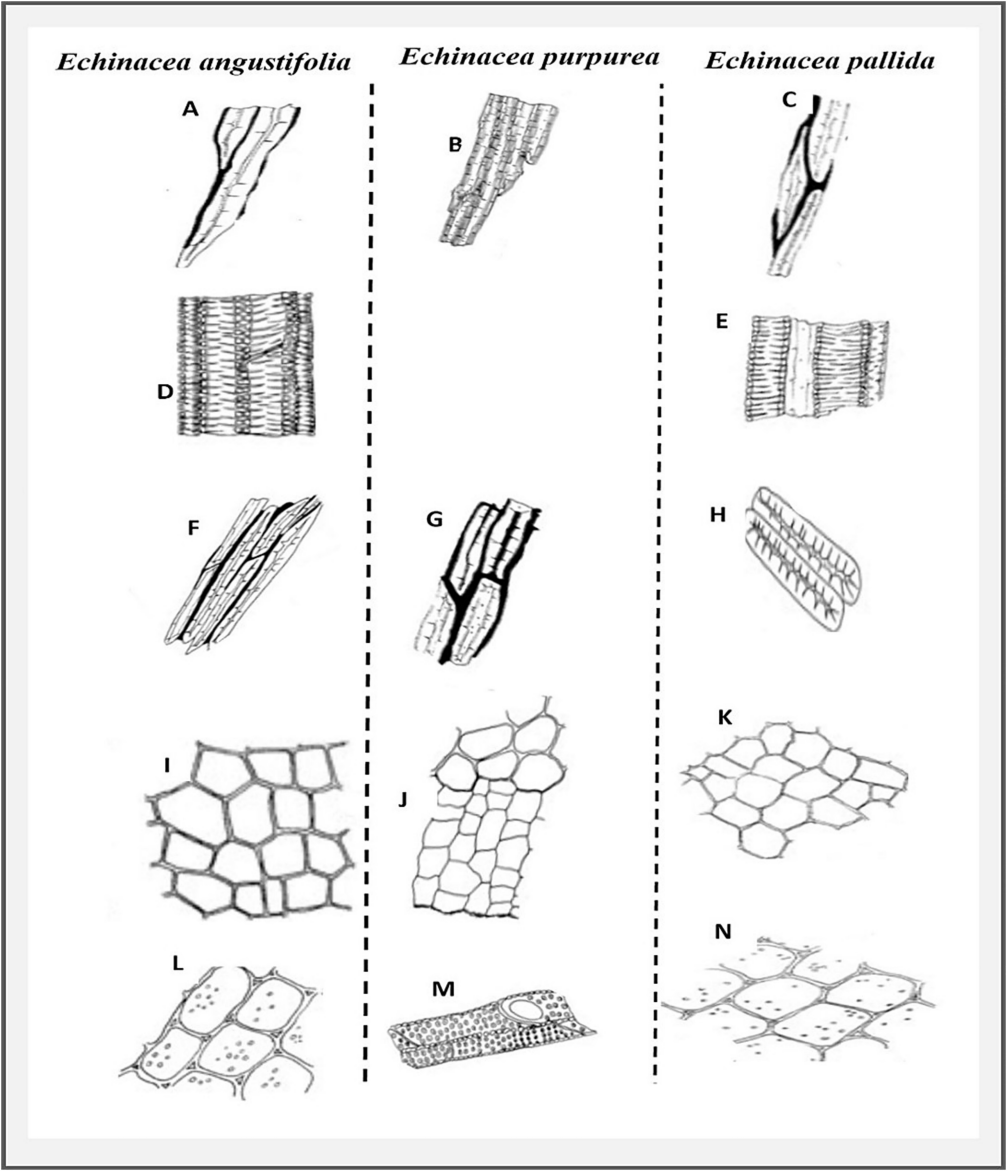
Illustration of microscopic diagnostic features in *Echinacea* species, adapted from the British Pharmacopoeia monograph [55] (A) Narrow lignified fibers with black phyto‐melanin in *E. angustifolia* . (B) Spindle‐shaped fibers in *E. purpurea* . (C) Lignified fibers with black phyto‐melanin in *E. pallida* . (D) Vessels with reticulate bordered‐pitted thickenings in *E. angustifolia* . (E) Vessels with reticulate thickenings in *E. pallida* . (F, G) Sclereids with phyto‐melanin in *E. angustifolia* and *E. pallida* respectively. (H) Sclereids with no phyto‐melanin in *E. purpurea* . (I, L) Outer layers with squarish and rectangular cells with fine wall pitted parenchyma in *E. angustifolia* . (J, M) Squarish and rectangular cells of outer layers, pitted in *E. purpurea* . (K, N) Squarish to rectangular cells with abundant thin‐walled and pitted parenchyma in *E. pallida* .

*Echinacea purpurea* is rich in phytochemicals with significant therapeutic potential. It exhibits a range of biological activities, including antioxidant, immunomodulatory, anti‐inflammatory, antibacterial, antiviral, and antiosteoporotic effects [52, 53]. These properties underscore its ecological and medicinal importance. Notably, the phytochemical composition and pharmacological effects can vary depending on the specific *Echinacea* species, leading to differences in the biological activities of their extracts [54]. Accurate identification of *Echinacea* species ( *Echinacea purpurea* , *Echinacea angustifolia* , and *Echinacea pallida* ) based on morphological traits is complicated by phenological variability within the species and overlapping visual characteristics [35, 54, 55, 56, 57, 58] (Figure 2). According to the British Pharmacopoeia monographs and other morphological features‐based studies, the roots of *E. purpurea* , *E. angustifolia* , and *E. pallida* exhibit similar macroscopic characteristics [55, 56]. All three species have cylindrical and spirally twisted roots that are longitudinally wrinkled with a light to reddish brown color. These species also exhibit several morphological similarities and their anatomical features. All three species possess lignified fibers arranged in long bundles and groups of squarish to rectangular cells in the outer root layers. Both *E. angustifolia* and *E. pallida* display black phyto‐melanin deposits associated with their fibers and sclereids, whereas *E. purpurea* lacks these deposits (Figure 2A–C). The vessels in *E. angustifolia* and *E. pallida* exhibit reticulate and scalariform thickenings, with *E. angustifolia* also showing bordered‐pitted thickenings (Figure 2D,E). All three species feature oil secretory canals. Sclereids are abundant in all three species; however, their shapes and the presence of phyto‐melanin deposits vary. *E. purpurea* sclereids are devoid of black phyto‐melanin, whereas *E. angustifolia* and *E. pallida* show these deposits in elongated to rectangular, and rectangular to irregular sclereids, respectively (Figure 2F–H). Fine‐walled, pitted parenchyma is abundant in all (Figure 2I–N). The absence of phyto‐melanin deposits in the sclereids of *E. purpurea* is the only distinct difference. However, this is not a reliable diagnostic feature because phyto‐melanin‐coated sclereids are present in the rhizome of the same species [59]. The remaining macroscopic and microscopic features are similar among these three species, making it difficult to accurately identify them using only morphological characteristics, particularly when samples are in powdered form.

#### 2.1.2 *Panax* (Ginseng)

*Panax quinquefolius* L. (American ginseng) is one of the most widely used medicinal herbs in the US and has been used in Chinese traditional medicines for thousands of years to treat cold and flu by reinforcing the immune system [60, 61]. Due to the high demand for ginseng products, American ginseng is adulterated with Asian ginseng ( *Panax ginseng* ) as both species share morphological and chemical characteristics [62, 63]. In both species, the roots are fusiform, cylindrical, and branched. Transverse sections of the roots show a wide outer zone with scattered reddish resin canals. Microscopically, fragments of parenchyma cells containing calcium oxalate are found in both species, along with fragments of large secretory canals containing resin in granular masses. These similarities in the roots, both macroscopically and microscopically, complicate the accurate authentication of the ginseng products [64, 65].

#### 2.1.3 Radix *Angelica Sinensis*

Radix *Angelica sinensis* (Danggui), a Chinese herb root, is commonly used in treating gynecological conditions [66, 67, 68]. Besides its medical use, Danggui is also used by women worldwide as a health food supplement [69]. Its identification becomes challenging when processed and incorporated into Chinese patent medicines [70, 71]. Due to high demand, many adulterants have emerged on the market, including species from the same genus and different genera, owing to morphological similarities [72, 73]. This issue has been highlighted in numerous studies focused on Danggui [71, 74, 75, 76, 77, 78]. However, all potential adulterants are not comprehensively covered in the British Pharmacopoeia. Here, we discuss two species of the genus *Angelica* and their morphological similarities [78]. According to the British Pharmacopoeia monographs*, Angelica sinensis* roots are yellowish brown, whereas *Angelica archangelica* roots are reddish brown. The root powder of both species ranges from yellowish to reddish with brown fragments of cork. The cork in both species is longitudinally wrinkled. Additionally, the powder from both species also contains small groups of single starch granules. Most features are similar when diagnostic tests are performed using morphological traits, making accurate identification crucial to ensure the authenticity and efficacy of medicinal preparations [77].

#### 2.1.4 Chamomile

Chamomile is widely used as a sedative, anxiolytic, antispasmodic, and treatment for mild skin irritation and inflammation [79, 80, 81]. There are numerous forms of chamomile, with the two most popular being *Matricaria recutita* (German chamomile) and *Anthemis nobilis* (Roman chamomile). German chamomile is one of the oldest and most extensively utilized plants globally [81]. It is considered more potent than Roman chamomile, has received more scientific evaluation, and is more widely cultivated [81, 82, 83]. *Matricaria recutita* is prone to adulteration with morphologically similar species such as *Anthemis nobilis* , *Anthemis cotula* , and *Senecio* species. *Senecio* species are reported to be hepatotoxic due to their pyrrolizidine alkaloid content [84], and *A. cotula* is known to be toxic due to its high content of coumarins and the sesquiterpene lactone anthecotulide, which can cause vomiting, diarrhea, and allergic reactions [85]. *A. nobilis* , although not toxic, is less effective than *Matricaria recutita* [84].

Below are the similarities between German and Roman chamomile as documented in the British Pharmacopoeia [86]. Both species possess capitula, which consist of an involucre made up of numerous bracts arranged in one to three rows. The capitula features marginal ligulate florets that are white, surrounding central yellow tubular florets. The involucral bracts have scarious margins. The inferior ovary is dark brown and is accompanied by a long style and a bifid stigma. The tubular florets have a five‐toothed corolla tube, five syngenesious, epipetalous stamens, and a gynoecium similar to the ligulate florets. Microscopically, the outer epidermis of the involucral bracts in both species consists of anomocytic stomata and glandular trichomes. Fragments of the involucral bracts also contain finely pitted sclereid cells at the base. The outer epidermis of ligulate florets is covered with striated cuticle and papillose cells at the apex of the florets. Fragments of the base of the flower, where the ovary is located, exhibit a ring of thick‐walled sclerenchymatous cells. Parenchyma cells of the ovary and involucral bracts contain small clusters of calcium oxalate crystals. Groups of cells at the apex of the stigma form elongated papillae. Pollen grains are spherical or triangular with germinal pores and spiny exine. These detailed macroscopic and microscopic characteristics, present in both species, complicate accurate authentication using only morphological features.

Relying solely on morphological features for identifying plants faces several challenges. Intraspecies variability can lead to significant phenological differences within the same species, making accurate identification difficult. Interspecies similarities further complicate the process, as different species may share similar morphological traits, increasing the risk of misidentification [52, 53, 54, 55, 56, 57, 58, 59, 60, 61, 62, 63, 64, 65, 66, 67, 68, 69, 70, 71, 72, 73, 74, 75, 76, 77, 78, 79, 80, 81, 82, 83, 84, 85, 86]. During initial harvesting, a lack of key features, such as flowers, poses a problem when plants are harvested for parts like roots that do not exhibit these identifying characteristics. The critical issue here is the accurate identification of plants and the determination of the optimal harvesting stage to ensure a high yield of the desired compounds. If the wrong species is harvested due to morphological similarities with the target species or if the plant is harvested at a stage when it is not producing the desired compounds—which are not always marker compounds of the species—it becomes challenging to identify the mistake during later stages of processing. Thus, proper identification and timing of harvest are essential to avoid such issues [87, 88]. Additionally, in herbal medicine, the processing of plant materials into powders or other forms can obscure critical morphological traits, making identification even more challenging [89, 90]. Even with advanced automated plant species identification tools such as digital cameras and mobile devices, morphological descriptors [91, 92, 93] alone are insufficient to address the issue when plant material is available in powdered form.

Consequently, these limitations in morphological identification can lead to the intentional or unintentional adulteration of plant materials during the supply chain, a problem that has been repeatedly reported in various cases by the American Botanical Council [94]. This often results in the final product being either devoid of or inferior in its chemical and therapeutic properties. In such cases, chemical/molecular methods are recommended as additional methods for ensuring accurate authentication and safeguarding consumers' health.

## 3 Chemical Methods for Herbal Plant Authentication: Quality Control and Authenticity

Chemical methods are widely used and conventionally suggested as quality control techniques and are recognized by major pharmacopeias worldwide, including the United States Pharmacopeia and National Formulary, the European Pharmacopeia, the British Pharmacopoeia, the Pharmacopoeia of the People's Republic of China, the Japan Pharmacopoeia, the Indian Pharmacopoeia, and the Korea Pharmacopoeia [36, 95, 96, 97]. These pharmacopeias recommend the utilization of different chromatographic, spectrometric, and spectroscopic techniques for the qualitative and quantitative assessments of herbal materials in domestic and international trade [36]. The chemical tests outlined in monographs serve a dual role in herbal medicine: They help identify the plant species by detecting specific markers and evaluate quality by confirming the presence of bioactive compounds that contribute to the therapeutic effects.

Up to date, the use of simple chromatographic techniques like TLC, HPTLC, HPLC, and GC has been more common in the industry due to the cost‐effectiveness, high throughput, and simple operation protocols [12, 33]. These techniques have proven effective for identifying plant part substitutions, which are common in commercially important medicinal plants like *Hypericum perforatum* and *Withania somnifera* , as well as species adulteration such as the adulteration of *Curcuma longa* (turmeric) with its wild relative [26, 42, 43]. In addition to chromatographic fingerprinting, spectroscopic (such as NMR, UV, IR, and NIR), spectrometry (such as MS), and hyphenated techniques (LC‐MS, HPLC‐UV, and GC‐MC) are also utilized in the herbal plant industry to enhance the accuracy of adulteration detection [33, 34, 65].

Before the advent of chemical fingerprinting, the widely used method for the authentication of herbal plants and herbal medicinal products (HMPs) was a component‐based approach, which focused on the identification of one or a few targeted markers [98, 99, 100]. This approach is preferred when the chemical properties of specific compounds are well‐characterized. However, since the therapeutic effects of medicinal plants are often the result of combined action of multiple chemical constituents, relying solely on single characteristic markers is often insufficient for accurate authentication [101]. A major limitation of using standard markers for authentication is that many bioactive compounds are not unique to a single species. For example, scutellarin occurs in various species of *Scutellaria* (Lamiaceae) and *Erigeron* (Asteraceae) [102, 103], whereas berberine is found in plants from the *Berberis* genus (Berberidaceae) as well as *Mahonia*, *Coptis*, and *Phellodendron*, spanning different families [101, 104, 105]. This lack of species specificity reduces their effectiveness as “standard markers” for precise species‐level identification. Accurate authentication requires species‐specific chemical markers, such as coptisine for *Coptis* species [105]. Additionally, the primary bioactive compound may not always be present at every stage of herbal material processing, potentially leading to false negatives, where materials are wrongly rejected due to the marker's absence [106, 107].

Unlike the component‐based approach, which targets a few specific markers, fingerprinting provides a detailed chemical profile of the entire plant material, making it more effective in detecting adulteration, substitutions, and variations in plant materials, even when specific markers may be absent or insufficient. This method allows for better species discrimination, particularly in cases where closely related species may look similar but have distinct chemical compositions and pharmacological effects. For instance, in the case of *Echinacea*, a popular immune‐boosting plant, different species within the genus often share morphological similarities, leading to potential confusion. However, each *Echinacea* species has unique pharmacological properties and distinct phytochemical profiles. Both HPLC and HPTLC fingerprinting methods have proven to be effective in ensuring the quality and safety of Echinacea products by distinguishing between these species [57]. *Echinacea purpurea* (the time‐tested species) has a characteristic presence of chicoric acid, caftaric acid, and chlorogenic acid with a minimal amount or absence of cyanarin, echinacoside, and alkamides, the other two *Echinacea* species, *E. augustifolia* and *E. pallida* , possess echinacoside as their major component. These compound variations were detected using HPLC‐CAD (charged aerosol detector) and advanced HPTLC techniques [57]. However, the chemical composition of *E. purpurea* can be significantly affected by the timing of harvest or the growth stage, posing a challenge when identifying the species using only chemical methods [108].

The advancements in chemical fingerprinting techniques contribute to the reliable authentication and quality control of herbal plants and HMPs. It is important to note that chemical fingerprinting has its limitations. For certain herbs, specific chemical markers remain unidentified. Additionally, there are cases where adulterants and substitutes share the same chemical profile, complicating identification. Variability in chemical profiles at different growth stages can further challenge the accuracy of results. Moreover, processing methods may alter or degrade chemical compounds, making the interpretation of the data more complex [101, 102, 103, 104, 105, 108]. Other issues are the nonuniformity in certification methods via chemical analyses, including varying choices of mobile and stationary phases in TLC and HPLC, the differing result interpretations, and the minimal concentration of active compounds that vary in different pharmacopeias worldwide. Therefore, there is still the need to establish a unified set of guidelines for chemical analysis of botanical species. Despite these limitations, chemical methods remain valuable when used alongside other authentication techniques, such as morphological analysis, to improve the reliability and accuracy of plant species authentication [109]. However, they may still fall short in some cases. For instance, in *Glycyrrhiza* species (licorice) both chemical and morphological characters are insufficient for correct identification [110]. Therefore, molecular markers, such as DNA sequencing, are increasingly used for accurate identification of *Glycyrrhiza* species [111, 112]. In such cases, DNA‐based methods become essential, providing species‐level identification and ensuring the authenticity of herbal plants and products where both morphological and chemical approaches fall short.

## 4 DNA Barcoding: Advantages and Applications in Species Identification

Chemical profiling and morphological characters are not always sufficient for accurate taxonomic identification because chemical compounds can also differ due to many nongenetic factors, such as climate, time of harvest, and postharvesting processing and morphology are not helpful if the plant part lacks the key characteristic or are provided as powdered or processed plant products [113, 114]. The development of numerous molecular methods that produce molecular markers has made it feasible to recognize plants with accuracy. These methods either take advantage of variations at the level of DNA or the protein that it codes for. Therefore, when other methods are insufficient, a molecular test is the most effective approach for overcoming the challenges of traditional taxonomy [115, 116, 117, 118, 119].

### 4.1 Established Methods for DNA Fingerprinting and DNA Barcoding

Various molecular approaches, such as DNA fingerprinting (also known as DNA profiling), have been developed to investigate genetic diversity in plants [99]. DNA fingerprinting involves analyzing the size differences of DNA fragments. Restriction fragment length polymorphism (RFLP) [120], random amplified polymorphic DNA (RAPD) [121], amplified fragment length polymorphism (AFLP) [122], simple sequence repeats (SSR) [123], and inter–simple sequence repeats (ISSR) [124] are other techniques used in this context [125]. The advantages of fingerprinting include low cost and the absence of a need for a reference sequence of the target species. However, DNA fingerprinting is generally less effective for plant authentication due to the high level of genetic variability and complexity within plant species.

DNA barcoding and genome sequencing are powerful tools in molecular biology, each suited to different purposes and applications. DNA barcoding involves sequencing a short, standardized region of the genome for species identification, making it a rapid, cost‐effective, and simple method ideal for taxonomy and industrial settings such as food quality control and environmental monitoring [16, 17, 126, 127, 128, 129, 130, 131]. DNA barcoding can investigate a single region or multiregion sequences [132]. In contrast, genome sequencing provides comprehensive genetic information by determining the complete DNA sequence of an organism [133, 134, 135, 136]. Genome sequencing is more suitable for primary research, clinical applications, and detailed genetic studies due to its depth of information and resolution, requiring significant computational resources and expertise.

Compared to other identification methods, DNA barcoding offers several advantages. It enables standardized identification for various fields such as biomedicine, agriculture, environmental testing, and endangered species management [97, 137, 138, 139, 140]. Additionally, practical applications of DNA barcoding include biosecurity, disease vector monitoring, law enforcement, and primatology [141, 142, 143]. Taxonomists can usually recognize the majority of organisms they are familiar with, but a constantly expanding community needs taxonomic data for a wide variety of taxa. DNA barcoding also alleviates the identification burden on taxonomists, allowing them to focus on defining taxa, resolving relationships, and discovering new species [140, 144]. Thus, DNA barcoding, widely regarded as a groundbreaking taxonomic tool, may be the most reliable framework currently available for classifying specimens and specimen‐based data in systematic research [145]. To support this, various databases of DNA barcode sequences have been established, with GenBank and the Barcode of Life Data System (BOLD) providing key repositories for these sequences [146]. Other well‐established databases include the Chinese Herbal Medicine DNA Barcode Identification System [147] and the DNA Barcode Research Center in Canada (CCDB) [148]. For plants, a sequence dataset of the nuclear ribosomal internal transcribed spacer (PLANiTS) has also been established [149].

In DNA barcoding, sequences of DNA amplicons are compared to a reference library for species identification, but this process can be challenging if reference sequences are unavailable. The method's accuracy and effectiveness rely heavily on the availability and comprehensiveness of these reference libraries. However, to enhance the robustness and reliability of DNA barcoding, there is a pressing need for expanding even more reference libraries. By increasing the number of species represented and the quality of the reference sequences, we can improve the precision of species identification. The Darwin Tree of Life Project [150] is already making significant strides in this area, contributing with valuable sequences to the existing libraries/databases. The British Pharmacopoeia catalogs DNA barcodes for several species prone to adulteration. For example, *Tribulus terrestris* , a commonly traded stimulant and food additive in Europe and the USA, is often substituted with other species within the same genus. *Anethum graveolens* is frequently used as an adulterant for *Foeniculum vulgare* . Similarly, *Glehnia littoralis* , a wild and threatened species, is frequently substituted with unrelated species. *Ocimum tenuiflorum* (Tulsi) also faces adulteration issues, with substitutions from other species within its genus [151, 152, 153, 154, 155].

DNA barcoding uses standard genetic markers or “barcoding regions” that can be used as single‐locus barcodes and multiple‐locus barcodes [156, 157, 158, 159]. The *maturase* K (*mat*K), *large subunit of ribulose 1,5 bisphosphate carboxylase* (*rbc*L), intergenic spacer region of *trn*H and photosystem II protein D1(*trn*H‐*psb*A), and internal transcribed spacer (ITS) are some of the commonly used single locus barcodes [114]. Numerous plastid locus combinations, such as *rbc*L + *psb*A‐*trn*H [160], polymerase gamma subunit *rpo*C1 + polymerase β subunit (*rpo*B) + *mat*K or *rpo*C1 + *mat*K + *trn*H‐*psb*A [161], and *mat*K + *ATP synthase subunit b‐delta* (*atp*F‐H) + *psb*K‐*psb*I or *mat*K + *atp*F‐H + *trn*H‐*psb*A have been proposed as multi‐locus barcodes [11, 162], when a single locus was found to be insufficient for discriminating between plants.

DNA barcoding is also useful in cases involving cryptic species and phenotypic plasticity. It offers a cost‐effective, faster, and more accurate method for species identification and can be used at any life stage. For a description of methodologies, refer to the papers by Sgamma *et al*. [97] and Howard *et al*. [140].

### 4.2 Recent Developments in Plant DNA Barcoding

DNA barcoding has become a crucial tool for species identification, with recent advancements significantly enhancing its effectiveness and applications in plant taxonomy. Notable developments include high‐resolution melting (HRM) curve analysis combined with barcoding (Bar‐HRM), DNA mini‐barcoding, and DNA meta‐barcoding [24, 163, 164, 165, 166, 167].

HRM technology has been combined with DNA barcoding, resulting in the development of Bar‐HRM technology [123, 163]. The principle behind HRM is based on monitoring the change in fluorescence of a double‐stranded DNA‐binding dye as the temperature is increased. As the temperature rises, the double‐stranded DNA denatures (separates into single strands). When this process happens, the fluorescence decreases. The temperature at which half of the DNA molecules are denatured is called the melting temperature (Tm). By monitoring the change in fluorescence, HRM can detect differences in the melting behavior which is characteristic of nucleic acid sequences [168]. The fluorescence of the DNA‐binding dye is continuously monitored in real time using a specialized instrument called real‐time PCR machine. The data obtained from HRM are analyzed using specialized software that analyses the shape of the melting curves and identifies any differences or mutations present in the samples. This analysis is usually qualitative (detecting the presence or absence of mutations). This approach does not require sequence‐specific probes, enabling the utilization of various DNA barcodes such as *rbc*L, *mat*K, *trn*H‐*psb*A, *rpo*C, and ITS for species identification [164]. There are, of course, some limitations to this approach, such as the potential difficulty in detecting closely related species with minimal genetic variability. Additionally, the strategy must be tailored to each plant or species, requiring the design of specific primers based on available sequences. Factors like amplicon size, G/C content, and primer design are also crucial for the accurate and reliable detection of nucleic acid sequence variations. Despite this, numerous studies have provided evidence for the efficacy of combining HRM analysis with DNA barcoding in the identification of various plant species, including those of significant economic value and those utilized in traditional medicine [164, 169, 170, 171]. For instance, Zhang *et al*. [172] successfully employed HRM analysis to distinguish between different species of ginseng (*Panax* spp.), renowned for their medicinal properties. Additionally, other studies showed how Bar‐HRM can be used with herbal products to detect low amounts of toxic medicinal materials in mixed samples [172, 173]. For instance, Singtonat and Osathanunkul [174] show the detection of the hepatotoxic *Crotalaria spectabilis* in *Thunbergia laurifolia* products using four BAR‐HRM primers designed on *rbcL*, *rpoC*, and *trnL*. Another study showed that BAR‐HRM was effective in identifying *Hebanthe eriantha* and *Pfaffia glomerata*, which are commonly grouped under the name “Brazilian Ginseng” [173].

When working with herbal products one of the main challenges is the extraction of good‐quality DNA as this can be degraded during the manufacturing process, resulting in failed amplification for standard barcodes [175]. In cases where full‐length barcodes may deteriorate, shorter versions of barcodes, known as mini‐barcodes, are utilized [33, 103, 123, 140, 163, 176, 177, 178, 179]. Therefore, DNA mini‐barcoding targeting and amplifying smaller DNA segments overcomes the issue of DNA degradation. Mini‐barcodes typically amplify 200 base pairs or less in length and can be amplified more quickly than standard barcodes. Due to their shorter amplicon length, mini‐barcodes exhibit a higher success rate in PCR amplification [130, 180]. However, the length limitation of mini‐barcodes becomes a challenge when a natural herbal product contains more species. Additionally, assembling a single sequence from mini‐barcode fragments can be difficult, and the presence of volatile mutation spots further complicates the identification process. Nevertheless, mini‐barcodes have proven valuable for differentiating closely related species using nucleotide signatures and stable single nucleotide polymorphism (SNP) locations. Although DNA mini‐barcoding offers certain advantages, it also has some drawbacks that need to be considered, including challenges in certain closely related species differentiation, the absence of a reference database, multiplexing difficulties, and limited phylogenetic information [175, 181].

An example displaying the use of mini‐barcodes for differentiating closely related plant species using stable SNP locations is orchids. Orchids are known for their high species diversity and challenging taxonomy due to subtle morphological differences. Mini‐barcodes, such as the ITS2 region, have been successfully used to distinguish closely related orchid species, facilitating conservation efforts and identifying potential new species [182]. Similarly, differentiating between species of ginseng, such as *P. ginseng* (Asian ginseng) and *P. quinquefolius* (American ginseng), can be challenging due to their morphological similarities [49, 65]. Mini‐barcodes combined with SNP analysis have been employed to accurately distinguish valuable medicinal herbs [183, 184, 185]. The use of mini‐barcodes and SNP analysis has proven to be effective in resolving taxonomic uncertainties and providing valuable insights for conservation, medicinal, and agricultural purposes.

All the above molecular techniques are subject to practical limitations as they mostly apply to single‐ingredient herbal preparations. DNA meta‐barcoding has emerged as a powerful technique to analyze complex samples containing DNA of different origins using High Throughput Sequencing methods [167, 186, 187, 188]. Meta‐barcoding allows for the simultaneous amplification of multiple DNA barcodes using universal PCR primers, enabling the analysis of species richness and composition in environmental samples [189, 190, 191]. This technique has facilitated ecological studies, including investigations into community composition changes in response to environmental factors [192, 193]. However, DNA meta‐barcoding presents challenges in taxonomic classification [194, 195]. Assigning correct taxonomic names to collected DNA sequences can be complex, especially when considering variations in amplified fragment length. Additionally, meta‐barcoding, like DNA barcoding, still needs to rely on the presence of quality reference databases on which to base identifications, and often, sequences deposited in GenBank are not rigorously checked [187, 196, 197]. Meta barcoding requires some specifications in terms of locus selection to capture species‐level resolution in different taxa targeting, for instance, multiple loci that will then be analyzed independently before being combined to produce a list of likely plants in the sample analyzed [197, 198]. Metabarcoding is also similar to the traditional DNA barcoding technique for the requirement of the whole barcode region to be present and not degraded.

DNA metabarcoding has been applied in the authentication of many herbal products. For instance, Frigerio *et al*. [199] used a multimarker approach to identify herbal tea composition. They analyzed 15 commercial tea products focusing on two barcode regions: the nuclear ITS2 and, the plastidial intergenic spacer *psbA‐trnH*. Additionally, they created six mock mixtures of plants in the laboratory starting both from raw plants (biomass) and genomic DNA (gDNA) to evaluate the quantitative ability of HTS. Howard *et al*. [176] used meta‐barcoding to detect *H. perforatum* DNA sequences in processed medicines. Out of 20 different matrices tested, the assay confirmed that *H. perforatum* was the major species in all positive samples, although trace contaminants were also detected. This study also highlighted another issue related to meta‐barcoding, that is, the need for high‐level analytical skills, which makes this technique more suitable for primary research rather than for industrial quality control. Furthermore, Raclariu *et al*. [186] stated that, although meta‐barcoding is a valid technique to identify each single species within complex multi‐ingredient and processed mixtures simultaneously when concerning quality control of herbal products, other techniques should be used to obtain quantitative and qualitative information of active metabolites. Therefore, from a pharmacognosy and pharmacovigilance point of view, a combination of analytical methods is the best route for products quality control products [186].

The United States Pharmacopeial Convention recommends the use of DNA barcoding for the identification of botanical species but emphasizes its conjunction with chemical or botanical methods. Industrial Quality Assurance/Quality Control (QA/QC) regulatory bodies often have limited choices for quality tests, such as molecular‐marked‐based tests like DNA barcoding and chemical analyses [97]. Among these methods, even DNA barcoding requires high‐quality DNA [171]. In many cases, degradation of DNA is unavoidable due to processing with large amounts of solvent and exposure to excessive heat and pressure, as HMP extracts aim to concentrate the maximum amount of active medicinal plant principles. This can result in “false negatives.” Moreover, since HMPsare primarily used for their therapeutic effects, which are mediated by their chemical constituents, relying solely on DNA methods in QA/QC may not be sufficient, as they cannot determine or certify the qualitative and quantitative chemical profile. Additionally, substitution with unauthorized plant parts is a concerning/prevailing practice in the HMP industry, presenting a potential threat to consumers due to variations in content and overall pharmacological activity of the medicinal plant parts. This issue remains unaddressed when DNA barcoding is the sole method of testing.

## 5 The Complexity of Authenticating HMPS and the Potential of an Orthogonal Approach

**FIGURE 3:**
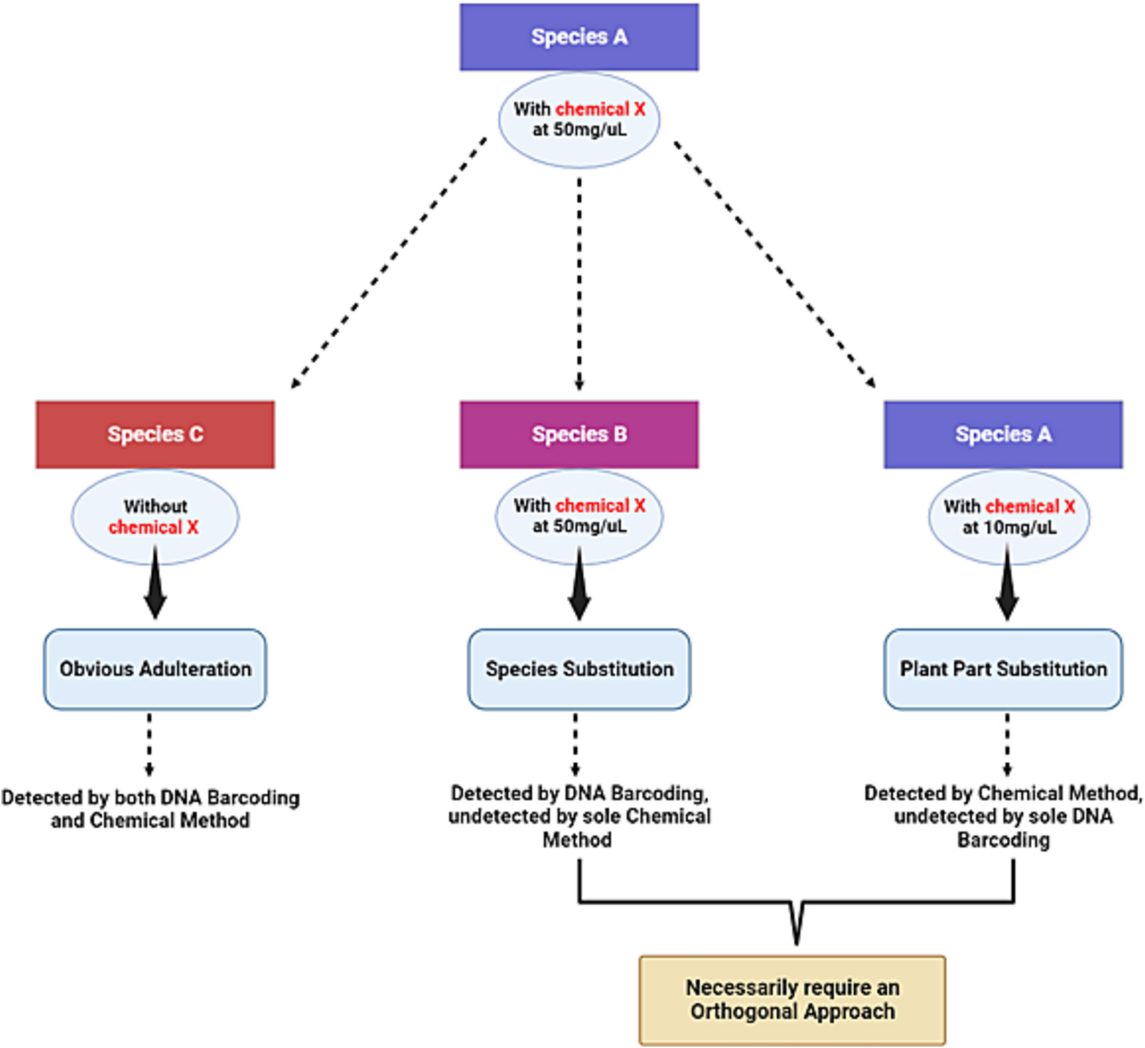
An exemplary situation of whether and when an orthogonal approach is needed.

**FIGURE 4:**
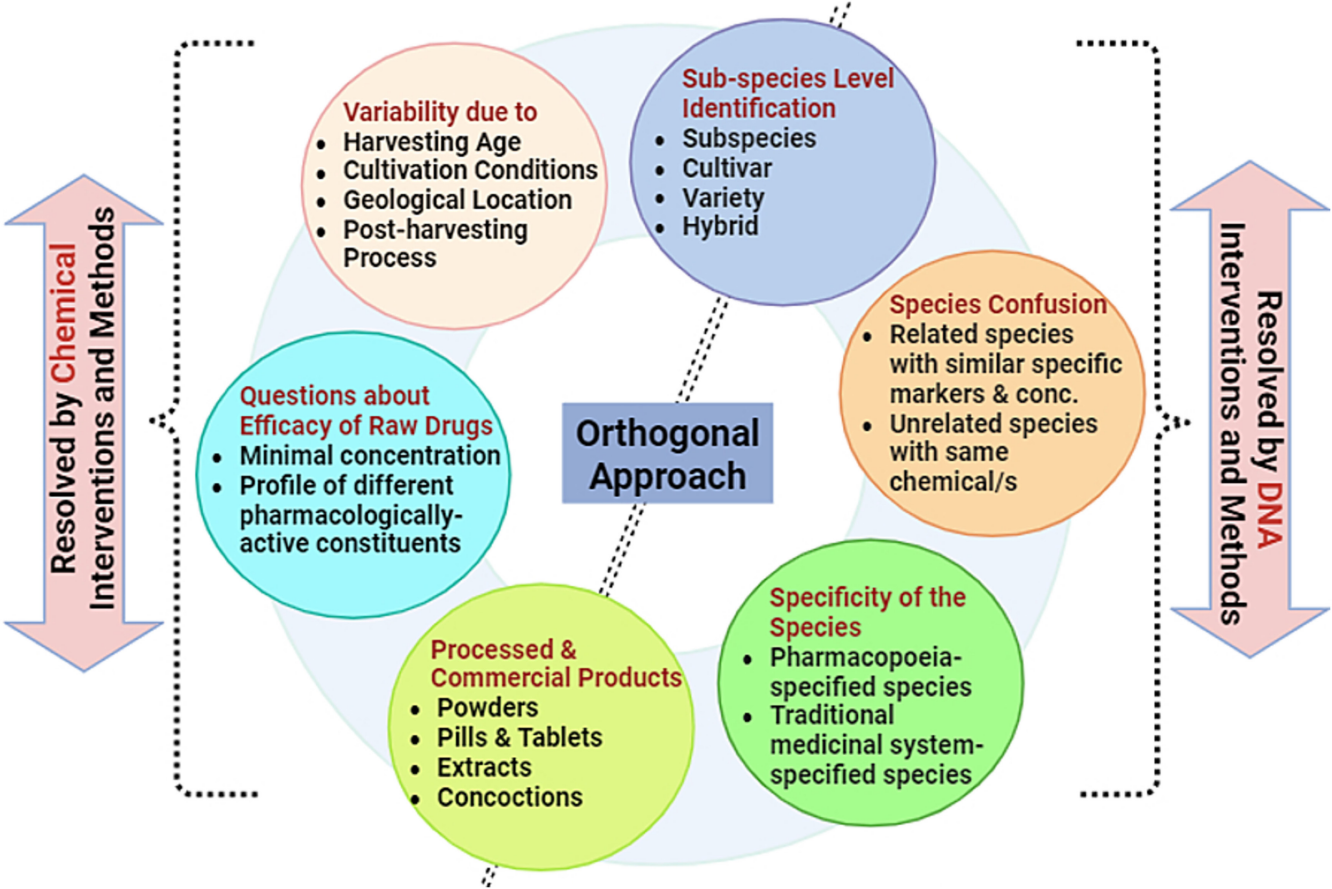
Complementary scenario where genetic (DNA) and chemical‐based interventions and methods help in an orthogonal approach of herbal medicinal plants and HMP authentication. (Conc. = concentration).

DNA barcoding has shown promise as an effective tool for identifying plant species, it is not without its limitations, especially when it comes to detecting adulteration. To ensure a robust and comprehensive authentication process, a combination of diverse techniques is imperative (Figure 3). In recent years, researchers and experts have emphasized the necessity of adopting an orthogonal approach toward HMP authentication [28, 29, 44, 200, 201, 202, 203, 204]. One such approach is the integration of chemical testing methods, including chromatography, spectrometry, and spectroscopy, which provide a broader scope by assessing the pharmacologically active compounds within a plant [205]. Although DNA barcoding is highly effective at confirming species identity, especially in the early stages of the screening process, it can be compromised by extensive product processing, substitution with different plant parts, or harvesting at an improper growth stage [206]. In contrast, chemical analyses focus on the quality and quantity of bioactive components, ensuring the plant's therapeutic potential. However, chemical testing alone may fail to reveal the true botanical identity if the substituted species contains similar compounds as shown in Figure 3. By using different methods, a comprehensive and reliable authentication regime is established, ensuring the botanical and chemical integrity of herbal products (Figure 4). This dual approach is particularly important in cases of species substitution, as some incorrect species may still contain minimal amounts of active chemical markers, leading to misleading results (Figure 3).

The works of Palhares *et al*. [205, 206], Sgamma *et al*. [97], and Raclariu *et al*. [186] have underlined the importance of employing multiple techniques to achieve accurate results. Studies listed in Table 2 illustrate where the synergy between DNA barcoding and chemical fingerprinting methods is used for plant authentication. Additionally, scenarios are discussed below where orthogonal approaches are effectively utilized.

**TABLE 2:** Utilization of orthogonal approaches for authenticating HMPs and products in various studies.

Species name	Part used	Barcode loci and DNA technique	Analytical technique/s	Reference
**DNA barcoding and chromatography techniques**				
*Hamamelis virginiana*	Leaves	*matK*, *rbcL*, and ITS2	TLC and HPLC	Palhares *et al*. [205]
*Matricariarecutita*	Flowers			
*Maytenusilicifolia*	Leaves			
*Mikania glomerata*	Leaves			
*Panax ginseng*	Roots			
*Passiflora incarnata*	Leaves			
*Peumus boldus*	Leaves			
*Valeriana officinalis*	Roots			
*Cinchona* species	Bark	*rbcL* and *matK*	HPLC	Palhares *et al*. [206]
*Angelica dahurica*	Roots	ITS	GC	Tabanca *et al*. [207]
*Angelica pubescentis*	Roots	ITS	GC	Tabanca *et al*. [207]
*Phellodendron*species	Bark	*trnH‐psbA* and ITS	HPLC	Zhang *et al*. [208]
*Curcuma longa*	Rhizomes	*rbcL*, *matK*, and ITS	HPLC	Duan *et al*. [209]
*Glycyrrhiza* species	Rhizomes and roots	ITS and *trnH‐psbA*	HPTLC	Frommenwiler *et al*. [210]
*Eurycoma longifolia*	Roots	*rbcL* and ITS2	HPLC	Abubakar *et al*. [211]
*Panax notoginseng*	Roots	ITS2	HPLC	Yang *et al*. [212]
*Panax vietnamensis*var*. Fuscidicus*	Roots			
*Echinacea* species	Roots	ITS1, ITS2, and metabarcoding	HPTLC	Raclariu *et al*. [213]
*Aristolochia* species	Roots	*rbcL*, *matK*, *ITS2*, and *trnH‐psbA*	HPTLC	Dechbumroong *et al*. [214]
*Galphimia glauca*	Leaves	*matK*, *rbcL*, *rpoC1*, *psbA‐trnH*, ITS1, and ITS2	TLC	Gesto‐Borroto *et al*. [215]
*Pueraria montana* var. *lobata*	Roots	ITS2	TLC, HPLC	Zhang *et al*. [216]
*Cyanthillium cinereum*	Whole plant	*rbcL*, *matK*, *ITS*, and *psbA‐trnH*	HPTLC	Thongkhao *et al*. [217]
*Arnebia decumbens*	Roots	ITS2	HPLC	Xu *et al*. [218]
*Arnebiaeuchroma*	Roots			
*Arnebia guttata*	Roots			
*Mallotusrepandus*	Stem	*rbcL*, *matK*, ITS, *trnH‐psbA*, and Bar‐HRM	HPTLC	Thongkhao *et al*. [219]
*Matricariarecutita*	Flowers	*rbcL*, *matK*, *trnH‐psbA*, ITS, and ITS2	HPTLC	Mahgoub *et al*. [81]
*Mucuna* species	Seeds	ITS2	HPLC	Intharuksa *et al*. [220]
**DNA barcoding and spectroscopy techniques**				
*Crataegus* species	Leaves	*rbcL*, *matK*, *psbA‐trnH*, and ITS2	NMR spectroscopy	Zarrei *et al*. [221]
*Saracaasoca*	Bark	*psbA‐trnH* and *rbcL*	NMR spectroscopy	Urumarudappa *et al*. [222]
*Hippophae* species	Fruits	ITS2 and *trnH‐psbA*	NMR spectroscopy	Liu *et al*. [223]
*Garcinia* species	Fruits	ITS, *psbA‐trnH*, and *rbcL*	NMR spectroscopy	Seethapathy *et al*. [224]
*Periploca indica*	Roots	*rbcL*, *matK*, and genome skimming	NMR spectroscopy	Kesanakurti *et al*. [225]
*Smilax* species	Roots			
*Atractylodes* species	Rhizomes	ITS	NMR spectroscopy	Shirahata *et al*. [226]
*Rauvolfia caffra*	Bark	*rbcL* and *matK*	NMR spectroscopy	Chipiti *et al*. [227]
**DNA barcoding and spectrometry techniques**				
*Echinacea purpurea*	Different parts	*rbcL* (Sanger sequencing), *ITS2* (Sanger + NGS)	Q‐TOF‐MS (coupled with HPLC)	Ivanova *et al*. [228]
*Valeriana officinalis*				
*Ginkgo biloba*				
*Hypericum perforatum*				
*Trigonella foenum‐graecum*				
*Vanilla* species	Seeds pods	*psaB* gene (not to be confused with *psbA*)	Stable isotope ratio mass spectrometry (sIRMS)	Geißler *et al*. [229]
*Citrus reticulata*	Dried Pericarp	ITS2	Q‐TOF‐MS (coupled with LC)	Li *et al*. [230]
*Lepidium meyenii*	Roots	*trnL*, ITS2, *psbA*, and *matK*	Flow injection mass spectrometry (FIMS)	Geng *et al*. [231]
**DNA barcoding and hyphenated techniques or combinations**				
*Cistanchedeserticola*	Succulent stems	ITS and ITS2	UPLC‐QTOF‐MS	Zheng *et al*. [232]
*Cistanchetubulosa*	Succulent stems			
*Ocimumbasilicum*	Leaves	*rbcL* and *matK*	GC‐MS	Elansary and Mahmoud [233]
*Aristolochiaceae* family members	Different parts	ITS2, *psbA‐trnH* (+ RT‐PCR assay)	UHPLC‐HR‐MS	Dechbumroong *et al*. [214]
*Actaea racemosa*	Root, underground stem	ITS and *trnH‐psbA*	MS and NMR	Harnly *et al*. [234]
*Rhodiola* species	Root	ITS2	HPTLC and NMR	Booker *et al*. [235]
*Veronica officinalis*	Aerial parts	ITS, ITS2, and metabarcoding	HPLC‐MS	Raclariu *et al*. [236]
*H. perforatum*	Flowers and leaves	ITS and metabarcoding	TLC and HPLC‐MS	Raclariu *et al*. [237]
*Glycyrrhiza* species	Roots	ITS and *trnV‐ndhC*	1‐D, 2‐D NMR, LC/UV, and LC/MS/MS	Song *et al*. [238]
*Artemisia absinthium*	Mixed part	*matK*, *rbcL*, *trnL*/IGS–intergenic spacer, *trnL‐trnF*, *ycf1*, ITS1, and ITS2	GC–MS	Paranaiba *et al*. [239]
*Stephania* species	Root and rhizomes	ITS2	HPLC‐QTOF‐MS/MS, and UHPLC‐DAD	Zhao *et al*. [240]
*Asarum* species	Root and rhizomes	ITS2	GC‐MS	Yao *et al*. [241]
*Gentiana macrophylla*	Rhizomes	ITS2	TOF‐MS and NMR	Li *et al*. [242]
*Gentiana straminea*	Rhizomes			
*Gentiana crassicaulis*	Rhizomes			
*Gentiana dahurica*	Rhizomes			
*Salvia* subg*. Perovskia*	Roots and leaves	*trnH‐psbA* and ITS2	UHPLC‐QTOF‐MS	Bielecka *et al*. [243]
*Echinacea* species	Root	Metabarcoding and genome skim	HPLC‐UV	Handy *et al*. [244]
*Erythroxylum*species	Bark and leaves	*rbcL*, *matK*, and *trnH‐psbA*	GC‐MS and NMR	Alberts and Meyer [245]
*Crocus sativus*	Stigma	*rbcL*	GC‐MS and ATR‐FTIR spectroscopy	Naim *et al*. [246]
*Trillium govanianum*	Rhizomes	*rbcL*, *matK*, ITS, and *trnH‐psbA*	HPTLC, LC‐MS, and NMR	Kumar *et al*. [247]

### 5.1 Species Traceability at the Subspecies Level

Studies have shown that this approach is also effective when species traceability is complicated by adulterants or substitutes at the subspecies level, such as cultivars or varieties. For instance, although HPLC fingerprinting failed to differentiate between species of *Phellodendron* (e.g., *P. amurense* , *P. chinense* , and *P. chinense* var. *glabriusculum*) at the variety level, DNA barcoding using markers such as *trn*H‐*psb*A and ITS successfully distinguished between these botanical species commonly used in Traditional Chinese Medicine (TCM) [208].

### 5.2 Addressing Discrepancies in Pharmacopeias

Another important aspect of utilizing this approach is its significance at a global level, where only specific species or varieties are listed in national pharmacopeias. For instance, whereas Korean, Chinese, and European pharmacopeias regard all three commercial species of *Glycyrrhiza* (*G. glabra, G. uralensis*, and *G. inflata* ) as licorice and use them in a replaceable manner, United States and Japanese pharmacopeias do not accept *G. inflata* as medicinal licorice and Indian pharmacopeia and traditional Indian medicinal systems like Ayurveda regard only *G. glabra* as medicinal species [203]. In such a scenario, species identity becomes integral, even when the chemical constitution is relatively similar. The labels in international marketspaces usually display only the popular name (licorice) or the binomial names without being checked by any botanical authority, the chances of species substitution increase significantly. However, employing the orthogonal approach in the analysis of 28 commercially available licorice samples revealed that HPTLC fingerprints had limited success in distinguishing the three species. In contrast, DNA barcoding effectively differentiated them based on distinct genotypes, utilizing well‐established plant barcodes like *trn*H‐*psb*A and ITS [210]. The safety net provided by DNA barcoding enhances the effectiveness of HPLC and HPTLC fingerprints in plant identification, establishing a clear and discrete authentication regime required for the concerned medicinal species [203]. Studies show that more advanced hyphenated methods like ultra‐high‐performance liquid chromatography (UHPLC)–UV show higher complementarity with DNA barcoding as they discriminate the three confusing species of *Glycyrrhiza* based on species‐specific metabolites and relative abundance of chalcones and flavones, proving the superiority of the orthogonal approach [208, 210].

### 5.3 Plant Identification Issues Arising From Migration and Globalization

The globalization of plants also involves the migration of seeds and plants, as well as the traditional knowledge of indigenous medicinal plants that accompanies the migration of people. These migrations in the last century have not only posed challenges for societies but have also exposed discrepancies between traditional and scientific nomenclature, which often go unnoticed [16, 154]. Such inconsistencies can create significant issues for quality control and consumer protection in importing countries. For example, seeds and plants of *Ocimum tenuiflorium* were brought to the UK from Africa and India. During this migration, *O*. *tenuiflorium* was replaced by *O. gratissimum* , leading to the cultivation of *O. gratissimum* by South Asian communities to alleviate symptoms related to Type 2 Diabetes. A study conducted at De Montfort University addressed this issue by developing DNA barcodes for *O*. *tenuiflorium* to distinguish it from other *Ocimum* species. This effort was further enhanced by incorporating chemical fingerprinting and morphological features, showcasing the successful application of an orthogonal approach to resolving identification challenges in herbal medicine [16, 154, 178].

Additionally, it is important to note that the orthogonal approach is most successful when carefully designed. When selecting chemical markers for analysis, several key points need to be considered regarding their specificity—whether they are unique to a particular species or common across a broader range of plants. It is essential to apply both qualitative and quantitative assessments to even specific chemical markers to ensure the bioactivity of the active components, which is the primary reason for using and consuming herbal plants and products. Furthermore, the complementarity of chemical methods with DNA barcoding depends on the specific objectives of the analysis. For instance, when evaluating raw drugs that claim to contain a specific chemical component, such as quinine (an antimalarial alkaloid) in *Cinchona*, a straightforward TLC and/or HPLC assay may be sufficient [248]. In contrast, when investigating the source of essential oils, such as those from *Angelica dahurica* and *Angelica pubescentis*, GC would serve as a better complement to DNA barcoding due to its ability to assess volatile compounds within the oils [207]. Thus, careful consideration of the specificity of markers, the application of both qualitative and quantitative assessments, and alignment of analytical methods with research objectives are critical for effective analysis.

The orthogonal approach should not be confined to industrial settings. Heinrich *et al*. [28, 29] emphasized that to ensure reproducibility and accurate interpretations of pharmacological, toxicological, and clinical/intervention studies involving botanicals, researchers should provide a botanical and morphological description of the starting botanical or herbal extracts used, alongside other chemical tests. Moreover, orthogonal approaches can be particularly valuable in resolving identification issues of plants that have migrated with people, ensuring that traditional and medicinal uses of these plants are accurately preserved and correctly identified in new environments. The Society for Medicinal Plant and Natural Product Research has established detailed requirements for the scientific study of medicinal plants, emphasizing the need for accurate identification and thorough documentation of plant starting materials. This guidance is designed not only for researchers but also for reviewers and editors assessing research for publication, ensuring that the findings are scientifically sound and reproducible [249].

In this review, we emphasize the importance of establishing more stringent benchmarks for plant identification. Given the persistent challenges in ensuring the quality, purity, and safety of plant materials, it is crucial to continually reassess and enhance authentication and quality assurance techniques. DNA‐based methods offer a valuable complement or alternative to traditional approaches, particularly when other methods prove unreliable. Implementing orthogonal strategies—such as combining chemical, botanical, morphological, and DNA‐based techniques—can significantly strengthen quality assurance, safeguarding the integrity of plant‐based research and products. Relying on a plethora of techniques, each providing unique insights, is essential to overcoming the limitations of any singular approach. By embracing an orthogonal approach that combines DNA barcoding, chemical fingerprinting, and other relevant authentication methods, we can achieve more accurate identification, ensure product quality, and support reliable and robust research outcomes in herbal medicine.

## Data Availability

The paper is a review of the literature as described in the introduction of the text. The original data used for making tables, figures, analysing data, discussion and conclusion is presented in the reference part of the manuscript.
